# Correction: Assessing the feasibility of large language models to identify top research priorities in enhanced external counterpulsation

**DOI:** 10.1371/journal.pone.0336055

**Published:** 2025-11-03

**Authors:** Shengkun Gai, Fangwan Huang, Xuanyun Liu, Ryan G. Benton, Glen M. Borchert, Jingshan Huang, Xiuyu Leng

There are errors in the author affiliations. The correct affiliations are as follows:

Shengkun Gai^1,2^, Fangwan Huang^3^, Xuanyun Liu^3^, Ryan G. Benton4, Glen M. Borchert^5^, Jingshan Huang^6^, Xiuyu Leng^2,7^

**1** Department of Cardiology, Linfen People’s Hospital, Linfen, Shanxi, China, **2** Department of Cardiology, The First Affiliated Hospital, Sun Yat-sen University, Guangzhou, China, **3** College of Computer and Data Science, Fuzhou University, Fuzhou, China, **4** School of Computing, University of South Alabama, Mobile, Alabama, United States of America, **5** College of Medicine, University of South Alabama, Mobile, Alabama, United States of America, **6** School of Computing and College of Medicine, University of South Alabama, Mobile, Alabama, United States of America, **7** NHC Key Laboratory of Assisted Circulation and Vascular Diseases (Sun Yat-sen University), Guangzhou, China
