# Supplementary material for: Assessing the feasibility of large language models to identify top research priorities in enhanced external counterpulsation
Source: PLoS One. 2025 Apr 15;20(4):e0305442. doi: 10.1371/journal.pone.0305442 (PMC11999140; doi:10.1371/journal.pone.0305442)
Supplement: S1 File — (ZIP) [file pone.0305442.s001.zip › raw data and results --- ERNIE Botts 2.docx]

**应用大数据语言模型确定体外反搏研究重点”\n---- -ERNIE Bot**

**Mechanisms**

体外反搏的机制 [量表题]

**本题平均分：**1

| 选项 | 小计 | 比例 |
| --- | --- | --- |
| 1 | 2 | 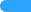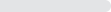22.22% |
| (空) | 7 | 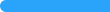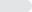77.78% |
| 本题有效填写人次 | 9 |  |

Study on the impact of enhanced external counterpulsation on hemodynamics and collateral vessel development.

增强型体外反搏对血流动力学及侧支血管发育的影响研究 [矩阵量表题]

**该矩阵题平均分：4.25**

| 题目\选项 | 1 | 2 | 3 | 4 | 5 | 平均分 |
| --- | --- | --- | --- | --- | --- | --- |
| relevance | 0(0%) | 0(0%) | 0(0%) | 2(22.22%) | 7(77.78%) | 4.78 |
| originality | 0(0%) | 1(11.11%) | 2(22.22%) | 3(33.33%) | 3(33.33%) | 3.89 |
| clarity | 0(0%) | 0(0%) | 1(11.11%) | 4(44.44%) | 4(44.44%) | 4.33 |
| specificity | 0(0%) | 0(0%) | 3(33.33%) | 3(33.33%) | 3(33.33%) | 4 |
| 小计 | 0(0%) | 1(2.78%) | 6(16.67%) | 12(33.33%) | 17(47.22%) | 4.25 |

Molecular mechanisms regulating endothelial cell function and smooth muscle cell behavior in EECP.

EECP调控内皮细胞功能和平滑肌细胞行为的分子机制研究 [矩阵量表题]

**该矩阵题平均分：4**

| 题目\选项 | 1 | 2 | 3 | 4 | 5 | 平均分 |
| --- | --- | --- | --- | --- | --- | --- |
| relevance | 0(0%) | 0(0%) | 0(0%) | 3(33.33%) | 6(66.67%) | 4.67 |
| originality | 0(0%) | 1(11.11%) | 1(11.11%) | 5(55.56%) | 2(22.22%) | 3.89 |
| clarity | 0(0%) | 0(0%) | 3(33.33%) | 5(55.56%) | 1(11.11%) | 3.78 |
| specificity | 0(0%) | 0(0%) | 4(44.44%) | 4(44.44%) | 1(11.11%) | 3.67 |
| 小计 | 0(0%) | 1(2.78%) | 8(22.22%) | 17(47.22%) | 10(27.78%) | 4 |

Exploration of the mechanism of enhanced external counterpulsation based on genomics.

基于基因组学的增强型体外反搏作用机制探索 [矩阵量表题]

**该矩阵题平均分：3.61**

| 题目\选项 | 1 | 2 | 3 | 4 | 5 | 平均分 |
| --- | --- | --- | --- | --- | --- | --- |
| relevance | 0(0%) | 1(11.11%) | 1(11.11%) | 4(44.44%) | 3(33.33%) | 4 |
| originality | 0(0%) | 3(33.33%) | 0(0%) | 3(33.33%) | 3(33.33%) | 3.67 |
| clarity | 0(0%) | 3(33.33%) | 0(0%) | 5(55.56%) | 1(11.11%) | 3.44 |
| specificity | 0(0%) | 3(33.33%) | 1(11.11%) | 4(44.44%) | 1(11.11%) | 3.33 |
| 小计 | 0(0%) | 10(27.78%) | 2(5.56%) | 16(44.44%) | 8(22.22%) | 3.61 |

Application of real-time imaging techniques in assessing the therapeutic effects of enhanced external counterpulsation

实时影像学技术在评估增强型体外反搏治疗效果中的应用研究 [矩阵量表题]

**该矩阵题平均分：3.86**

| 题目\选项 | 1 | 2 | 3 | 4 | 5 | 平均分 |
| --- | --- | --- | --- | --- | --- | --- |
| relevance | 0(0%) | 0(0%) | 3(33.33%) | 2(22.22%) | 4(44.44%) | 4.11 |
| originality | 0(0%) | 0(0%) | 4(44.44%) | 2(22.22%) | 3(33.33%) | 3.89 |
| clarity | 0(0%) | 0(0%) | 3(33.33%) | 5(55.56%) | 1(11.11%) | 3.78 |
| specificity | 0(0%) | 0(0%) | 4(44.44%) | 4(44.44%) | 1(11.11%) | 3.67 |
| 小计 | 0(0%) | 0(0%) | 14(38.89%) | 13(36.11%) | 9(25%) | 3.86 |

Comparative study of the efficacy of enhanced external counterpulsation and traditional drug therapy in cardiovascular and cerebrovascular diseases。

增强型体外反搏与传统药物治疗在心脑血管疾病中的疗效对比研究 [矩阵量表题]

**该矩阵题平均分：3.42**

| 题目\选项 | 1 | 2 | 3 | 4 | 5 | 平均分 |
| --- | --- | --- | --- | --- | --- | --- |
| relevance | 1(11.11%) | 0(0%) | 2(22.22%) | 3(33.33%) | 3(33.33%) | 3.78 |
| originality | 1(11.11%) | 1(11.11%) | 2(22.22%) | 4(44.44%) | 1(11.11%) | 3.33 |
| clarity | 1(11.11%) | 0(0%) | 3(33.33%) | 5(55.56%) | 0(0%) | 3.33 |
| specificity | 1(11.11%) | 0(0%) | 4(44.44%) | 4(44.44%) | 0(0%) | 3.22 |
| 小计 | 4(11.11%) | 1(2.78%) | 11(30.56%) | 16(44.44%) | 4(11.11%) | 3.42 |

Device improvements

结构改良 [量表题]

**本题平均分：**1

| 选项 | 小计 | 比例 |
| --- | --- | --- |
| 选项1 | 1 | 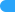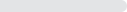11.11% |
| (空) | 8 | 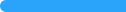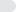88.89% |
| 本题有效填写人次 | 9 |  |

Application of intelligent perception and adaptive adjustment technology in enhanced external counterpulsation machines.

智能感知与自适应调节技术在增强型体外反搏机器中的应用研究 [矩阵量表题]

**该矩阵题平均分：4.19**

| 题目\选项 | 1 | 2 | 3 | 4 | 5 | 平均分 |
| --- | --- | --- | --- | --- | --- | --- |
| relevance | 0(0%) | 0(0%) | 1(11.11%) | 3(33.33%) | 5(55.56%) | 4.44 |
| originality | 0(0%) | 0(0%) | 1(11.11%) | 2(22.22%) | 6(66.67%) | 4.56 |
| clarity | 0(0%) | 0(0%) | 3(33.33%) | 5(55.56%) | 1(11.11%) | 3.78 |
| specificity | 0(0%) | 0(0%) | 2(22.22%) | 5(55.56%) | 2(22.22%) | 4 |
| 小计 | 0(0%) | 0(0%) | 7(19.44%) | 15(41.67%) | 14(38.89%) | 4.19 |

Optimization design of enhanced external counterpulsation machines based on biomechanical simulation.

基于生物力学仿真的增强型体外反搏机器优化设计 [矩阵量表题]

**该矩阵题平均分：4.11**

| 题目\选项 | 1 | 2 | 3 | 4 | 5 | 平均分 |
| --- | --- | --- | --- | --- | --- | --- |
| relevance | 0(0%) | 0(0%) | 1(11.11%) | 3(33.33%) | 5(55.56%) | 4.44 |
| originality | 0(0%) | 0(0%) | 1(11.11%) | 2(22.22%) | 6(66.67%) | 4.56 |
| clarity | 0(0%) | 1(11.11%) | 3(33.33%) | 3(33.33%) | 2(22.22%) | 3.67 |
| specificity | 0(0%) | 1(11.11%) | 2(22.22%) | 4(44.44%) | 2(22.22%) | 3.78 |
| 小计 | 0(0%) | 2(5.56%) | 7(19.44%) | 12(33.33%) | 15(41.67%) | 4.11 |

Research and application of new materials and technologies in enhanced external counterpulsation machines.

增强型体外反搏机器中新型材料与技术的研究与应用 [矩阵量表题]

**该矩阵题平均分：3.94**

| 题目\选项 | 1 | 2 | 3 | 4 | 5 | 平均分 |
| --- | --- | --- | --- | --- | --- | --- |
| relevance | 0(0%) | 0(0%) | 1(11.11%) | 4(44.44%) | 4(44.44%) | 4.33 |
| originality | 0(0%) | 0(0%) | 2(22.22%) | 5(55.56%) | 2(22.22%) | 4 |
| clarity | 0(0%) | 0(0%) | 3(33.33%) | 5(55.56%) | 1(11.11%) | 3.78 |
| specificity | 0(0%) | 0(0%) | 4(44.44%) | 4(44.44%) | 1(11.11%) | 3.67 |
| 小计 | 0(0%) | 0(0%) | 10(27.78%) | 18(50%) | 8(22.22%) | 3.94 |

Development and preliminary evaluation of wearable enhanced external counterpulsation devices.

可穿戴式增强型体外反搏设备的研发与初步应用评估 [矩阵量表题]

**该矩阵题平均分：3.56**

| 题目\选项 | 1 | 2 | 3 | 4 | 5 | 平均分 |
| --- | --- | --- | --- | --- | --- | --- |
| relevance | 0(0%) | 2(22.22%) | 1(11.11%) | 3(33.33%) | 3(33.33%) | 3.78 |
| originality | 0(0%) | 2(22.22%) | 2(22.22%) | 1(11.11%) | 4(44.44%) | 3.78 |
| clarity | 0(0%) | 3(33.33%) | 2(22.22%) | 2(22.22%) | 2(22.22%) | 3.33 |
| specificity | 0(0%) | 3(33.33%) | 1(11.11%) | 4(44.44%) | 1(11.11%) | 3.33 |
| 小计 | 0(0%) | 10(27.78%) | 6(16.67%) | 10(27.78%) | 10(27.78%) | 3.56 |

Research on multimodal feedback and control systems in enhanced external counterpulsation machines.

强型体外反搏机器中的多模态反馈与控制系统研究 [矩阵量表题]

**该矩阵题平均分：3.92**

| 题目\选项 | 1 | 2 | 3 | 4 | 5 | 平均分 |
| --- | --- | --- | --- | --- | --- | --- |
| relevance | 0(0%) | 0(0%) | 1(11.11%) | 4(44.44%) | 4(44.44%) | 4.33 |
| originality | 0(0%) | 0(0%) | 3(33.33%) | 3(33.33%) | 3(33.33%) | 4 |
| clarity | 0(0%) | 0(0%) | 4(44.44%) | 4(44.44%) | 1(11.11%) | 3.67 |
| specificity | 0(0%) | 0(0%) | 4(44.44%) | 4(44.44%) | 1(11.11%) | 3.67 |
| 小计 | 0(0%) | 0(0%) | 12(33.33%) | 15(41.67%) | 9(25%) | 3.92 |

In the field of heart disease

在心血管领域的应用 [量表题]

**本题平均分：**1

| 选项 | 小计 | 比例 |
| --- | --- | --- |
| 选项1 | 1 | 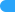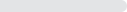11.11% |
| (空) | 8 | 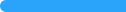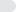88.89% |
| 本题有效填写人次 | 9 |  |

Effect prediction and optimization of enhanced external counterpulsation in cardiovascular treatment based on big data analysis.

基于大数据分析的增强型体外反搏在心脏内科治疗中的效果预测与优化 [矩阵量表题]

**该矩阵题平均分：4.31**

| 题目\选项 | 1 | 2 | 3 | 4 | 5 | 平均分 |
| --- | --- | --- | --- | --- | --- | --- |
| relevance | 0(0%) | 0(0%) | 1(11.11%) | 2(22.22%) | 6(66.67%) | 4.56 |
| originality | 0(0%) | 0(0%) | 2(22.22%) | 2(22.22%) | 5(55.56%) | 4.33 |
| clarity | 0(0%) | 0(0%) | 2(22.22%) | 4(44.44%) | 3(33.33%) | 4.11 |
| specificity | 0(0%) | 0(0%) | 1(11.11%) | 5(55.56%) | 3(33.33%) | 4.22 |
| 小计 | 0(0%) | 0(0%) | 6(16.67%) | 13(36.11%) | 17(47.22%) | 4.31 |

Exploration of the potential of enhanced external counterpulsation in post-heart transplant rehabilitation.

增强型体外反搏在心脏移植后康复中的潜力探索 [矩阵量表题]

**该矩阵题平均分：3.64**

| 题目\选项 | 1 | 2 | 3 | 4 | 5 | 平均分 |
| --- | --- | --- | --- | --- | --- | --- |
| relevance | 1(11.11%) | 0(0%) | 2(22.22%) | 2(22.22%) | 4(44.44%) | 3.89 |
| originality | 1(11.11%) | 0(0%) | 2(22.22%) | 2(22.22%) | 4(44.44%) | 3.89 |
| clarity | 1(11.11%) | 0(0%) | 4(44.44%) | 3(33.33%) | 1(11.11%) | 3.33 |
| specificity | 1(11.11%) | 0(0%) | 3(33.33%) | 4(44.44%) | 1(11.11%) | 3.44 |
| 小计 | 4(11.11%) | 0(0%) | 11(30.56%) | 11(30.56%) | 10(27.78%) | 3.64 |

Mechanism study using enhanced external counterpulsation to promote heart regeneration and repair.

利用增强型体外反搏技术促进心脏再生与修复的机理研究 [矩阵量表题]

**该矩阵题平均分：3.47**

| 题目\选项 | 1 | 2 | 3 | 4 | 5 | 平均分 |
| --- | --- | --- | --- | --- | --- | --- |
| relevance | 1(11.11%) | 0(0%) | 2(22.22%) | 3(33.33%) | 3(33.33%) | 3.78 |
| originality | 1(11.11%) | 0(0%) | 2(22.22%) | 2(22.22%) | 4(44.44%) | 3.89 |
| clarity | 1(11.11%) | 0(0%) | 5(55.56%) | 2(22.22%) | 1(11.11%) | 3.22 |
| specificity | 1(11.11%) | 1(11.11%) | 4(44.44%) | 3(33.33%) | 0(0%) | 3 |
| 小计 | 4(11.11%) | 1(2.78%) | 13(36.11%) | 10(27.78%) | 8(22.22%) | 3.47 |

Exploration of personalized treatment strategies using enhanced external counterpulsation in cardiovascular medicine

增强型体外反搏在心脏内科中的个性化治疗策略探索 [矩阵量表题]

**该矩阵题平均分：3.61**

| 题目\选项 | 1 | 2 | 3 | 4 | 5 | 平均分 |
| --- | --- | --- | --- | --- | --- | --- |
| relevance | 0(0%) | 1(11.11%) | 0(0%) | 4(44.44%) | 4(44.44%) | 4.22 |
| originality | 0(0%) | 2(22.22%) | 2(22.22%) | 3(33.33%) | 2(22.22%) | 3.56 |
| clarity | 0(0%) | 1(11.11%) | 3(33.33%) | 5(55.56%) | 0(0%) | 3.44 |
| specificity | 0(0%) | 2(22.22%) | 3(33.33%) | 4(44.44%) | 0(0%) | 3.22 |
| 小计 | 0(0%) | 6(16.67%) | 8(22.22%) | 16(44.44%) | 6(16.67%) | 3.61 |

Long-term monitoring and management research in cardiovascular medicine using wearable enhanced external counterpulsation technology.

基于可穿戴技术的增强型体外反搏心脏内科长期监测与管理研究 [矩阵量表题]

**该矩阵题平均分：3.36**

| 题目\选项 | 1 | 2 | 3 | 4 | 5 | 平均分 |
| --- | --- | --- | --- | --- | --- | --- |
| relevance | 1(11.11%) | 0(0%) | 1(11.11%) | 5(55.56%) | 2(22.22%) | 3.78 |
| originality | 1(11.11%) | 0(0%) | 2(22.22%) | 4(44.44%) | 2(22.22%) | 3.67 |
| clarity | 1(11.11%) | 2(22.22%) | 2(22.22%) | 4(44.44%) | 0(0%) | 3 |
| specificity | 1(11.11%) | 2(22.22%) | 2(22.22%) | 4(44.44%) | 0(0%) | 3 |
| 小计 | 4(11.11%) | 4(11.11%) | 7(19.44%) | 17(47.22%) | 4(11.11%) | 3.36 |

Applications in the field of neurology

在神经内科领域的应用 [量表题]

**本题平均分：**1

| 选项 | 小计 | 比例 |
| --- | --- | --- |
| 选项1 | 1 | 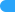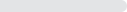11.11% |
| (空) | 8 | 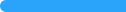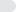88.89% |
| 本题有效填写人次 | 9 |  |

Mechanism of enhanced external counterpulsation in cerebral blood flow reconstruction in ischemic stroke patients.

增强型体外反搏在缺血性卒中患者脑血流重建中的作用机制 [矩阵量表题]

**该矩阵题平均分：3.86**

| 题目\选项 | 1 | 2 | 3 | 4 | 5 | 平均分 |
| --- | --- | --- | --- | --- | --- | --- |
| relevance | 0(0%) | 0(0%) | 1(11.11%) | 4(44.44%) | 4(44.44%) | 4.33 |
| originality | 0(0%) | 1(11.11%) | 2(22.22%) | 3(33.33%) | 3(33.33%) | 3.89 |
| clarity | 0(0%) | 0(0%) | 4(44.44%) | 4(44.44%) | 1(11.11%) | 3.67 |
| specificity | 0(0%) | 0(0%) | 5(55.56%) | 3(33.33%) | 1(11.11%) | 3.56 |
| 小计 | 0(0%) | 1(2.78%) | 12(33.33%) | 14(38.89%) | 9(25%) | 3.86 |

Evaluation of the cognitive function improvement effect of enhanced external counterpulsation based on neuroimaging.

基于神经影像学的增强型体外反搏对认知功能障碍改善效果的评估 [矩阵量表题]

**该矩阵题平均分：3.94**

| 题目\选项 | 1 | 2 | 3 | 4 | 5 | 平均分 |
| --- | --- | --- | --- | --- | --- | --- |
| relevance | 0(0%) | 0(0%) | 2(22.22%) | 3(33.33%) | 4(44.44%) | 4.22 |
| originality | 0(0%) | 0(0%) | 3(33.33%) | 3(33.33%) | 3(33.33%) | 4 |
| clarity | 0(0%) | 0(0%) | 3(33.33%) | 4(44.44%) | 2(22.22%) | 3.89 |
| specificity | 0(0%) | 1(11.11%) | 3(33.33%) | 3(33.33%) | 2(22.22%) | 3.67 |
| 小计 | 0(0%) | 1(2.78%) | 11(30.56%) | 13(36.11%) | 11(30.56%) | 3.94 |

Promotion of brain function reshaping in neurorehabilitation through enhanced external counterpulsation.

增强型体外反搏在神经康复中对脑功能重塑的促进作用 [矩阵量表题]

**该矩阵题平均分：3.97**

| 题目\选项 | 1 | 2 | 3 | 4 | 5 | 平均分 |
| --- | --- | --- | --- | --- | --- | --- |
| relevance | 0(0%) | 0(0%) | 1(11.11%) | 4(44.44%) | 4(44.44%) | 4.33 |
| originality | 0(0%) | 0(0%) | 2(22.22%) | 5(55.56%) | 2(22.22%) | 4 |
| clarity | 0(0%) | 0(0%) | 2(22.22%) | 6(66.67%) | 1(11.11%) | 3.89 |
| specificity | 0(0%) | 1(11.11%) | 2(22.22%) | 5(55.56%) | 1(11.11%) | 3.67 |
| 小计 | 0(0%) | 1(2.78%) | 7(19.44%) | 20(55.56%) | 8(22.22%) | 3.97 |

Prospective study using enhanced external counterpulsation to improve the quality of life in neuropathic pain patients.

利用增强型体外反搏技术改善神经疼痛患者生活质量的前瞻性研究 [矩阵量表题]

**该矩阵题平均分：3.39**

| 题目\选项 | 1 | 2 | 3 | 4 | 5 | 平均分 |
| --- | --- | --- | --- | --- | --- | --- |
| relevance | 1(11.11%) | 2(22.22%) | 0(0%) | 3(33.33%) | 3(33.33%) | 3.56 |
| originality | 1(11.11%) | 2(22.22%) | 1(11.11%) | 1(11.11%) | 4(44.44%) | 3.56 |
| clarity | 1(11.11%) | 2(22.22%) | 1(11.11%) | 3(33.33%) | 2(22.22%) | 3.33 |
| specificity | 1(11.11%) | 3(33.33%) | 1(11.11%) | 2(22.22%) | 2(22.22%) | 3.11 |
| 小计 | 4(11.11%) | 9(25%) | 3(8.33%) | 9(25%) | 11(30.56%) | 3.39 |

.Synchronization study of brain blood flow and neural activity using enhanced external counterpulsation technology.

利用增强型体外反搏技术促进脑血流与神经电活动的同步性研究 [矩阵量表题]

**该矩阵题平均分：4.08**

| 题目\选项 | 1 | 2 | 3 | 4 | 5 | 平均分 |
| --- | --- | --- | --- | --- | --- | --- |
| relevance | 0(0%) | 0(0%) | 1(11.11%) | 4(44.44%) | 4(44.44%) | 4.33 |
| originality | 0(0%) | 0(0%) | 2(22.22%) | 3(33.33%) | 4(44.44%) | 4.22 |
| clarity | 0(0%) | 0(0%) | 2(22.22%) | 5(55.56%) | 2(22.22%) | 4 |
| specificity | 0(0%) | 1(11.11%) | 2(22.22%) | 4(44.44%) | 2(22.22%) | 3.78 |
| 小计 | 0(0%) | 1(2.78%) | 7(19.44%) | 16(44.44%) | 12(33.33%) | 4.08 |

Applications in other fields

其他领域的应用 [量表题]

**本题平均分：**1

| 选项 | 小计 | 比例 |
| --- | --- | --- |
| 选项1 | 1 | 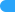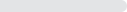11.11% |
| (空) | 8 | 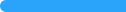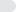88.89% |
| 本题有效填写人次 | 9 |  |

Exploration of the mechanism of skin regeneration and wound healing using enhanced external counterpulsation technology.

基于增强型体外反搏技术的皮肤再生与伤口愈合机制探索 [矩阵量表题]

**该矩阵题平均分：3.03**

| 题目\选项 | 1 | 2 | 3 | 4 | 5 | 平均分 |
| --- | --- | --- | --- | --- | --- | --- |
| relevance | 1(11.11%) | 3(33.33%) | 1(11.11%) | 3(33.33%) | 1(11.11%) | 3 |
| originality | 1(11.11%) | 2(22.22%) | 1(11.11%) | 2(22.22%) | 3(33.33%) | 3.44 |
| clarity | 1(11.11%) | 3(33.33%) | 2(22.22%) | 2(22.22%) | 1(11.11%) | 2.89 |
| specificity | 1(11.11%) | 4(44.44%) | 1(11.11%) | 2(22.22%) | 1(11.11%) | 2.78 |
| 小计 | 4(11.11%) | 12(33.33%) | 5(13.89%) | 9(25%) | 6(16.67%) | 3.03 |

Application of enhanced external counterpulsation in improving renal function in chronic kidney disease patients.

增强型体外反搏在改善慢性肾脏病患者肾功能中的应用研究 [矩阵量表题]

**该矩阵题平均分：3.47**

| 题目\选项 | 1 | 2 | 3 | 4 | 5 | 平均分 |
| --- | --- | --- | --- | --- | --- | --- |
| relevance | 0(0%) | 2(22.22%) | 1(11.11%) | 2(22.22%) | 4(44.44%) | 3.89 |
| originality | 0(0%) | 2(22.22%) | 1(11.11%) | 4(44.44%) | 2(22.22%) | 3.67 |
| clarity | 0(0%) | 2(22.22%) | 3(33.33%) | 4(44.44%) | 0(0%) | 3.22 |
| specificity | 0(0%) | 2(22.22%) | 4(44.44%) | 3(33.33%) | 0(0%) | 3.11 |
| 小计 | 0(0%) | 8(22.22%) | 9(25%) | 13(36.11%) | 6(16.67%) | 3.47 |

Promotion of healing and vascular regeneration in diabetic foot ulcers using enhanced external counterpulsation.

利用增强型体外反搏技术促进糖尿病足溃疡的愈合与血管再生 [矩阵量表题]

**该矩阵题平均分：3.75**

| 题目\选项 | 1 | 2 | 3 | 4 | 5 | 平均分 |
| --- | --- | --- | --- | --- | --- | --- |
| relevance | 0(0%) | 0(0%) | 2(22.22%) | 4(44.44%) | 3(33.33%) | 4.11 |
| originality | 0(0%) | 0(0%) | 4(44.44%) | 2(22.22%) | 3(33.33%) | 3.89 |
| clarity | 0(0%) | 0(0%) | 4(44.44%) | 5(55.56%) | 0(0%) | 3.56 |
| specificity | 0(0%) | 0(0%) | 5(55.56%) | 4(44.44%) | 0(0%) | 3.44 |
| 小计 | 0(0%) | 0(0%) | 15(41.67%) | 15(41.67%) | 6(16.67%) | 3.75 |

Potential study of enhanced external counterpulsation in improving joint blood flow and inflammation control in rheumatoid arthritis patients.

增强型体外反搏在改善类风湿性关节炎患者关节血流与炎症控制中的潜力研究

[矩阵量表题]

**该矩阵题平均分：3.5**

| 题目\选项 | 1 | 2 | 3 | 4 | 5 | 平均分 |
| --- | --- | --- | --- | --- | --- | --- |
| relevance | 1(11.11%) | 0(0%) | 3(33.33%) | 3(33.33%) | 2(22.22%) | 3.56 |
| originality | 1(11.11%) | 0(0%) | 2(22.22%) | 3(33.33%) | 3(33.33%) | 3.78 |
| clarity | 1(11.11%) | 0(0%) | 4(44.44%) | 2(22.22%) | 2(22.22%) | 3.44 |
| specificity | 1(11.11%) | 1(11.11%) | 3(33.33%) | 3(33.33%) | 1(11.11%) | 3.22 |
| 小计 | 4(11.11%) | 1(2.78%) | 12(33.33%) | 11(30.56%) | 8(22.22%) | 3.5 |

Role of enhanced external counterpulsation in promoting skin regeneration and reducing scar formation after burns.

增强型体外反搏在促进烧伤后皮肤再生与减少疤痕形成中的作用研究 [矩阵量表题]

**该矩阵题平均分：3.14**

| 题目\选项 | 1 | 2 | 3 | 4 | 5 | 平均分 |
| --- | --- | --- | --- | --- | --- | --- |
| relevance | 1(11.11%) | 3(33.33%) | 2(22.22%) | 2(22.22%) | 1(11.11%) | 2.89 |
| originality | 1(11.11%) | 1(11.11%) | 2(22.22%) | 1(11.11%) | 4(44.44%) | 3.67 |
| clarity | 1(11.11%) | 2(22.22%) | 2(22.22%) | 4(44.44%) | 0(0%) | 3 |
| specificity | 1(11.11%) | 1(11.11%) | 4(44.44%) | 3(33.33%) | 0(0%) | 3 |
| 小计 | 4(11.11%) | 7(19.44%) | 10(27.78%) | 10(27.78%) | 5(13.89%) | 3.14 |

**题目平均分之和：378.56**
