# Supplementary material for: Assessing the feasibility of large language models to identify top research priorities in enhanced external counterpulsation
Source: PLoS One. 2025 Apr 15;20(4):e0305442. doi: 10.1371/journal.pone.0305442 (PMC11999140; doi:10.1371/journal.pone.0305442)
Supplement: S1 File — (ZIP) [file pone.0305442.s001.zip › raw data and results --- ERNIE Botts.docx]

**应用大数据语言模型确定体外反搏研究重点”\n--- ERNIE Bot**

| 序号 | 1 | 2 | 3 | 4 | 5 | 6 | 7 | 8 | 9 |
| --- | --- | --- | --- | --- | --- | --- | --- | --- | --- |
| 提交答卷时间 | 2024/3/2 23:38:03 | 2024/3/3 15:06:02 | 2024/3/3 16:06:50 | 2024/3/3 17:01:44 | 2024/3/3 20:40:47 | 2024/3/3 21:25:21 | 2024/3/3 21:58:10 | 2024/3/3 22:51:15 | 2024/3/4 14:37:27 |
| 所用时间 | 875秒 | 955秒 | 278秒 | 277秒 | 460秒 | 439秒 | 416秒 | 463秒 | 258秒 |
| 来源 | 微信 | 微信 | 微信 | 微信 | 微信 | 微信 | 微信 | 微信 | 微信 |
| 来自IP | 125.94.200.112(广东-广州) | 113.128.150.106(山东-济南) | 101.224.155.104(上海-上海) | 58.63.138.219(广东-广州) | 117.147.112.183(浙江-杭州) | 118.249.59.8(湖南-长沙) | 123.123.46.248(北京-北京) | 120.229.30.215(广东-深圳) | 121.33.209.35(广东-广州) |
| **Mechanisms体外反搏的机制** |  |  |  |  |  |  |  |  |  |
| **1.relevance.** | 5 | 5 | 5 | 4 | 5 | 5 | 5 | 4 | 5 |
| **originality** | 4 | 3 | 5 | 4 | 3 | 2 | 5 | 4 | 5 |
| **clarity** | 3 | 5 | 5 | 4 | 5 | 4 | 4 | 4 | 5 |
| **specificity** | 3 | 5 | 5 | 3 | 4 | 3 | 4 | 4 | 5 |
| 2.**relevance** | 5 | 5 | 5 | 5 | 4 | 5 | 5 | 4 | 4 |
| **originality** | 4 | 3 | 5 | 4 | 4 | 2 | 5 | 4 | 4 |
| **clarity** | 3 | 4 | 5 | 4 | 4 | 3 | 4 | 4 | 3 |
| specificity | 3 | 4 | 5 | 3 | 4 | 3 | 4 | 4 | 3 |
| **3.relevance** | 5 | 5 | 5 | 4 | 4 | 4 | 4 | 2 | 3 |
| **originality** | 4 | 4 | 5 | 5 | 4 | 2 | 5 | 2 | 2 |
| **clarity** | 4 | 4 | 5 | 4 | 4 | 2 | 4 | 2 | 2 |
| **specificity** | 4 | 4 | 5 | 4 | 3 | 2 | 4 | 2 | 2 |
| **4.relevance** | 5 | 4 | 5 | 3 | 5 | 4 | 5 | 3 | 3 |
| **originality** | 5 | 4 | 5 | 3 | 4 | 3 | 5 | 3 | 3 |
| clarity | 4 | 4 | 5 | 3 | 4 | 4 | 4 | 3 | 3 |
| **specificity** | 4 | 4 | 5 | 3 | 4 | 3 | 4 | 3 | 3 |
| **5.relevance** | 4 | 3 | 5 | 4 | 5 | 3 | 5 | 1 | 4 |
| **originality** | 4 | 3 | 4 | 3 | 4 | 2 | 5 | 1 | 4 |
| **clarity** | 3 | 3 | 4 | 4 | 4 | 3 | 4 | 1 | 4 |
| **specificity** | 3 | 3 | 4 | 3 | 4 | 3 | 4 | 1 | 4 |
| **Device improvements 结构改良** |  |  |  |  |  |  |  |  |  |
| **1.relevance** | 5 | 5 | 5 | 4 | 5 | 4 | 5 | 3 | 4 |
| **originality** | 5 | 5 | 5 | 5 | 5 | 4 | 5 | 3 | 4 |
| **clarity** | 4 | 4 | 5 | 4 | 3 | 3 | 4 | 3 | 4 |
| **specificity** | 4 | 4 | 5 | 3 | 4 | 4 | 5 | 3 | 4 |
| **2.relevance** | 5 | 5 | 5 | 4 | 4 | 4 | 5 | 5 | 3 |
| originality | 5 | 5 | 5 | 5 | 4 | 4 | 5 | 5 | 3 |
| **clarity** | 4 | 4 | 5 | 3 | 2 | 3 | 4 | 5 | 3 |
| **specificity** | 4 | 4 | 4 | 4 | 2 | 3 | 5 | 5 | 3 |
| **3.relevance** | 5 | 5 | 5 | 4 | 4 | 4 | 5 | 4 | 3 |
| **originality** | 3 | 4 | 5 | 4 | 4 | 4 | 5 | 4 | 3 |
| **clarity** | 3 | 4 | 5 | 4 | 4 | 3 | 4 | 4 | 3 |
| **specificity** | 3 | 4 | 5 | 4 | 3 | 3 | 4 | 4 | 3 |
| **4.relevance** | 5 | 4 | 5 | 4 | 3 | 4 | 5 | 2 | 2 |
| **originality** | 5 | 4 | 5 | 5 | 3 | 3 | 5 | 2 | 2 |
| **clarity** | 3 | 4 | 5 | 4 | 2 | 3 | 5 | 2 | 2 |
| **specificity** | 3 | 4 | 5 | 4 | 2 | 4 | 4 | 2 | 2 |
| **5.relevance** | 5 | 5 | 5 | 4 | 4 | 4 | 5 | 4 | 3 |
| **originality** | 5 | 4 | 5 | 4 | 3 | 3 | 5 | 4 | 3 |
| **clarity** | 3 | 4 | 5 | 4 | 3 | 3 | 4 | 4 | 3 |
| **specificity** | 3 | 4 | 5 | 4 | 3 | 3 | 4 | 4 | 3 |
| **In the field of heart disease在心血管领域的应用** |  |  |  |  |  |  |  |  |  |
| **1.relevance** | 5 | 5 | 5 | 4 | 5 | 4 | 5 | 3 | 5 |
| **originality** | 5 | 5 | 5 | 4 | 4 | 3 | 5 | 3 | 5 |
| **clarity** | 4 | 5 | 5 | 4 | 4 | 3 | 4 | 3 | 5 |
| **specificity** | 4 | 5 | 5 | 4 | 4 | 4 | 4 | 3 | 5 |
| **2.relevance** | 5 | 4 | 5 | 5 | 3 | 4 | 5 | 1 | 3 |
| **originality** | 4 | 5 | 5 | 5 | 3 | 4 | 5 | 1 | 3 |
| **clarity** | 4 | 3 | 5 | 4 | 3 | 3 | 4 | 1 | 3 |
| **specificity** | 4 | 3 | 5 | 4 | 3 | 4 | 4 | 1 | 3 |
| **3.relevance** | 4 | 4 | 5 | 4 | 3 | 5 | 5 | 1 | 3 |
| **originality** | 4 | 5 | 5 | 5 | 4 | 3 | 5 | 1 | 3 |
| **clarity** | 3 | 3 | 4 | 5 | 3 | 3 | 4 | 1 | 3 |
| **specificity** | 3 | 3 | 4 | 4 | 3 | 2 | 4 | 1 | 3 |
| **4.relevance** | 5 | 5 | 5 | 4 | 4 | 4 | 5 | 2 | 4 |
| **originality** | 4 | 5 | 5 | 3 | 3 | 2 | 4 | 2 | 4 |
| **clarity** | 3 | 4 | 4 | 4 | 4 | 3 | 3 | 2 | 4 |
| **specificity** | 3 | 4 | 4 | 3 | 3 | 2 | 4 | 2 | 4 |
| **5.relevance** | 5 | 4 | 4 | 4 | 3 | 4 | 5 | 1 | 4 |
| **originality** | 4 | 4 | 5 | 4 | 3 | 3 | 5 | 1 | 4 |
| **clarity** | 3 | 4 | 4 | 3 | 2 | 2 | 4 | 1 | 4 |
| **specificity** | 3 | 4 | 4 | 3 | 2 | 2 | 4 | 1 | 4 |
| **Applications in the field of neurology在神经内科领域的应用** |  |  |  |  |  |  |  |  |  |
| **1.relevance** | 5 | 5 | 4 | 4 | 4 | 4 | 5 | 3 | 5 |
| **originality** | 4 | 4 | 5 | 4 | 3 | 2 | 5 | 3 | 5 |
| **clarity** | 3 | 4 | 4 | 4 | 3 | 3 | 4 | 3 | 5 |
| **specificity** | 3 | 4 | 4 | 3 | 3 | 3 | 4 | 3 | 5 |
| **2.relevance** | 5 | 5 | 5 | 4 | 4 | 4 | 5 | 3 | 3 |
| **originality** | 4 | 4 | 5 | 5 | 4 | 3 | 5 | 3 | 3 |
| **clarity** | 4 | 5 | 5 | 4 | 4 | 3 | 4 | 3 | 3 |
| **specificity** | 4 | 5 | 5 | 4 | 3 | 2 | 4 | 3 | 3 |
| **3.relevance** | 5 | 5 | 5 | 4 | 4 | 4 | 5 | 3 | 4 |
| **originality** | 4 | 4 | 5 | 4 | 4 | 3 | 5 | 3 | 4 |
| **clarity** | 4 | 4 | 5 | 4 | 4 | 3 | 4 | 3 | 4 |
| **specificity** | 4 | 4 | 5 | 4 | 3 | 2 | 4 | 3 | 4 |
| **4.relevance** | 4 | 5 | 5 | 4 | 2 | 4 | 5 | 1 | 2 |
| **originality** | 5 | 5 | 5 | 4 | 2 | 3 | 5 | 1 | 2 |
| **clarity** | 4 | 5 | 5 | 4 | 2 | 3 | 4 | 1 | 2 |
| **specificity** | 4 | 5 | 5 | 3 | 2 | 2 | 4 | 1 | 2 |
| **5.relevance** | 5 | 5 | 5 | 4 | 4 | 4 | 5 | 3 | 4 |
| **originality** | 5 | 5 | 5 | 4 | 4 | 3 | 5 | 3 | 4 |
| **clarity** | 5 | 4 | 5 | 4 | 4 | 3 | 4 | 3 | 4 |
| **specificity** | 5 | 4 | 5 | 4 | 3 | 2 | 4 | 3 | 4 |
| **Applications in other fields其他领域的应用** |  |  |  |  |  |  |  |  |  |
| **1.relevance** | 4 | 4 | 4 | 3 | 2 | 2 | 5 | 1 | 2 |
| **originality** | 5 | 4 | 5 | 4 | 2 | 3 | 5 | 1 | 2 |
| **clarity** | 4 | 3 | 5 | 3 | 2 | 2 | 4 | 1 | 2 |
| **specificity** | 4 | 3 | 5 | 2 | 2 | 2 | 4 | 1 | 2 |
| **2.relevance** | 5 | 5 | 5 | 3 | 4 | 4 | 5 | 2 | 2 |
| **originality** | 4 | 4 | 5 | 4 | 4 | 3 | 5 | 2 | 2 |
| **clarity** | 4 | 3 | 4 | 3 | 4 | 3 | 4 | 2 | 2 |
| **specificity** | 4 | 3 | 4 | 3 | 3 | 3 | 4 | 2 | 2 |
| **3.relevance** | 5 | 5 | 4 | 4 | 4 | 4 | 5 | 3 | 3 |
| **originality** | 4 | 5 | 5 | 4 | 3 | 3 | 5 | 3 | 3 |
| **clarity** | 4 | 4 | 4 | 4 | 3 | 3 | 4 | 3 | 3 |
| **specificity** | 3 | 4 | 4 | 4 | 3 | 3 | 4 | 3 | 3 |
| **4.relevance** | 5 | 4 | 4 | 3 | 3 | 4 | 5 | 1 | 3 |
| **originality** | 5 | 4 | 5 | 4 | 3 | 4 | 5 | 1 | 3 |
| **clarity** | 5 | 4 | 5 | 3 | 3 | 3 | 4 | 1 | 3 |
| **specificity** | 5 | 4 | 4 | 3 | 3 | 2 | 4 | 1 | 3 |
| **5.relevance** | 2 | 4 | 4 | 3 | 2 | 2 | 5 | 1 | 3 |
| **originality** | 5 | 5 | 5 | 4 | 2 | 3 | 5 | 1 | 3 |
| **clarity** | 4 | 4 | 4 | 3 | 2 | 2 | 4 | 1 | 3 |
| **specificity** | 3 | 4 | 4 | 3 | 2 | 3 | 4 | 1 | 3 |
| **Total score** | 410 | 420 | 475 | 381 | 337 | 314 | 455 | 244 | 332 |
