# Supplementary material for: Assessing the feasibility of large language models to identify top research priorities in enhanced external counterpulsation
Source: PLoS One. 2025 Apr 15;20(4):e0305442. doi: 10.1371/journal.pone.0305442 (PMC11999140; doi:10.1371/journal.pone.0305442)
Supplement: S1 File — (ZIP) [file pone.0305442.s001.zip › raw data and results --- ChatGPT .docx]

应用大数据语言模型确定体外反搏研究重点”\n--- ChatGPT

| 序号 | 1 | 2 | 3 | 4 | 5 | 6 | 7 | 8 | 9 |
| --- | --- | --- | --- | --- | --- | --- | --- | --- | --- |
| 提交答卷时间 | 2024/3/2 23:49:40 | 2024/3/3 15:22:21 | 2024/3/3 16:11:47 | 2024/3/3 17:05:15 | 2024/3/3 21:02:47 | 2024/3/3 21:44:08 | 2024/3/3 22:07:01 | 2024/3/3 22:57:51 | 2024/3/4 14:40:53 |
| 所用时间 | 680秒 | 975秒 | 292秒 | 205秒 | 1314秒 | 347秒 | 527秒 | 389秒 | 196秒 |
| 来源 | 微信 | 微信 | 微信 | 微信 | 微信 | 微信 | 微信 | 微信 | 微信 |
| 来源详情 | N/A | N/A | N/A | N/A | N/A | N/A | N/A | N/A | N/A |
| 来自IP | 125.94.200.112(广东-广州) | 113.128.150.106(山东-济南) | 101.224.155.104(上海-上海) | 58.63.138.219(广东-广州) | 117.147.112.183(浙江-杭州) | 118.249.59.8(湖南-长沙) | 123.123.46.248(北京-北京) | 120.229.30.215(广东-深圳) | 121.33.209.35(广东-广州) |
| the principles of enhanced external counterpulsation（体外反搏的机制） | -2 | -2 | -2 | -2 | -2 | -2 | 1 | -2 | -2 |
| 1.relevance | 5 | 5 | 5 | 4 | 5 | 4 | 5 | 2 | 3 |
| originality | 5 | 5 | 5 | 4 | 4 | 2 | 5 | 2 | 3 |
| clarity | 3 | 5 | 5 | 4 | 3 | 3 | 4 | 2 | 3 |
| specificity | 2 | 5 | 5 | 4 | 4 | 3 | 4 | 2 | 3 |
| 2.relevance | 4 | 4 | 5 | 3 | 4 | 4 | 5 | 2 | 3 |
| originality | 4 | 4 | 5 | 4 | 3 | 2 | 5 | 2 | 3 |
| clarity | 4 | 4 | 5 | 3 | 3 | 3 | 3 | 2 | 3 |
| specificity | 3 | 4 | 5 | 3 | 3 | 3 | 3 | 2 | 3 |
| 3.relevance | 5 | 3 | 5 | 4 | 4 | 4 | 5 | 2 | 4 |
| originality | 4 | 3 | 5 | 3 | 4 | 4 | 5 | 2 | 4 |
| clarity | 4 | 3 | 5 | 4 | 3 | 3 | 3 | 2 | 4 |
| specificity | 3 | 3 | 5 | 4 | 3 | 3 | 3 | 2 | 4 |
| 4.relevance | 5 | 3 | 5 | 5 | 3 | 4 | 5 | 2 | 3 |
| originality | 5 | 3 | 5 | 4 | 3 | 3 | 4 | 2 | 3 |
| clarity | 4 | 3 | 5 | 4 | 3 | 3 | 3 | 2 | 3 |
| specificity | 3 | 3 | 5 | 4 | 3 | 2 | 3 | 2 | 3 |
| 5.relevance | 5 | 5 | 5 | 4 | 4 | 4 | 5 | 3 | 3 |
| originality | 4 | 5 | 4 | 3 | 4 | 4 | 4 | 3 | 3 |
| clarity | 4 | 4 | 4 | 4 | 3 | 3 | 3 | 3 | 3 |
| specificity | 4 | 4 | 5 | 4 | 3 | 3 | 3 | 3 | 3 |
| Instrument improvements（结构改良） | -2 | -2 | -2 | -2 | -2 | -2 | 1 | -2 | -2 |
| 1.relevance | 4 | 5 | 4 | 3 | 3 | 4 | 5 | 1 | 2 |
| originality | 4 | 5 | 4 | 4 | 3 | 4 | 5 | 1 | 2 |
| clarity | 4 | 4 | 4 | 3 | 2 | 3 | 4 | 1 | 2 |
| specificity | 4 | 4 | 4 | 3 | 2 | 2 | 4 | 1 | 2 |
| 2.relevance | 5 | 5 | 4 | 3 | 3 | 4 | 5 | 2 | 2 |
| originality | 4 | 5 | 4 | 4 | 3 | 4 | 5 | 2 | 2 |
| clarity | 3 | 5 | 4 | 3 | 2 | 4 | 3 | 2 | 2 |
| specificity | 3 | 5 | 4 | 3 | 2 | 4 | 4 | 2 | 2 |
| 3.relevance | 5 | 4 | 4 | 4 | 4 | 4 | 5 | 3 | 4 |
| originality | 4 | 4 | 4 | 4 | 3 | 4 | 5 | 3 | 4 |
| clarity | 4 | 5 | 4 | 4 | 3 | 4 | 4 | 3 | 4 |
| specificity | 4 | 5 | 4 | 3 | 3 | 4 | 4 | 3 | 4 |
| 4.relevance | 5 | 5 | 4 | 3 | 3 | 4 | 5 | 3 | 3 |
| originality | 4 | 5 | 4 | 3 | 3 | 4 | 4 | 3 | 3 |
| clarity | 4 | 5 | 4 | 3 | 3 | 3 | 4 | 3 | 3 |
| specificity | 3 | 5 | 4 | 3 | 3 | 2 | 4 | 3 | 3 |
| 5.relevance | 5 | 5 | 4 | 3 | 3 | 4 | 5 | 1 | 4 |
| originality | 4 | 5 | 5 | 3 | 3 | 2 | 5 | 1 | 4 |
| clarity | 4 | 4 | 5 | 3 | 3 | 3 | 3 | 1 | 4 |
| specificity | 3 | 4 | 4 | 3 | 3 | 2 | 4 | 1 | 4 |
| in the field of heart disease（在心血管领域的应用） | -2 | -2 | -2 | -2 | -2 | -2 | 1 | -2 | -2 |
| 1.relevance | 5 | 3 | 4 | 4 | 4 | 4 | 5 | 3 | 3 |
| originality | 5 | 3 | 5 | 4 | 3 | 2 | 5 | 3 | 3 |
| clarity | 5 | 4 | 5 | 4 | 3 | 3 | 5 | 3 | 3 |
| specificity | 5 | 4 | 4 | 3 | 3 | 2 | 4 | 3 | 3 |
| 2.relevance | 3 | 5 | 4 | 4 | 3 | 4 | 5 | 3 | 3 |
| originality | 4 | 5 | 4 | 4 | 3 | 3 | 5 | 3 | 3 |
| clarity | 3 | 4 | 5 | 4 | 3 | 3 | 4 | 3 | 3 |
| specificity | 3 | 4 | 4 | 4 | 3 | 2 | 4 | 3 | 3 |
| 3.relevance | 5 | 4 | 4 | 4 | 4 | 4 | 5 | 2 | 4 |
| originality | 4 | 4 | 4 | 5 | 3 | 3 | 5 | 2 | 4 |
| clarity | 4 | 4 | 5 | 4 | 3 | 3 | 4 | 2 | 4 |
| specificity | 4 | 4 | 5 | 4 | 3 | 3 | 4 | 2 | 4 |
| 4.relevance | 5 | 5 | 4 | 4 | 4 | 4 | 5 | 3 | 3 |
| originality | 5 | 5 | 5 | 4 | 4 | 2 | 5 | 3 | 3 |
| clarity | 5 | 5 | 5 | 4 | 3 | 3 | 4 | 3 | 3 |
| specificity | 5 | 5 | 5 | 4 | 3 | 2 | 4 | 3 | 3 |
| 5.relevance | 5 | 5 | 4 | 4 | 3 | 4 | 5 | 1 | 3 |
| originality | 5 | 4 | 4 | 4 | 2 | 2 | 5 | 1 | 3 |
| clarity | 5 | 4 | 4 | 4 | 2 | 2 | 5 | 1 | 3 |
| specificity | 4 | 4 | 4 | 4 | 2 | 3 | 4 | 1 | 3 |
| in the field of neurology（在神经内科领域的应用） | -2 | -2 | -2 | -2 | -2 | -2 | 1 | -2 | -2 |
| 1.relevance | 5 | 5 | 4 | 4 | 4 | 4 | 5 | 3 | 3 |
| originality | 5 | 4 | 5 | 4 | 3 | 2 | 5 | 3 | 3 |
| clarity | 5 | 4 | 4 | 4 | 4 | 3 | 5 | 3 | 3 |
| specificity | 5 | 4 | 4 | 4 | 3 | 2 | 4 | 3 | 3 |
| 2.relevance | 5 | 3 | 4 | 4 | 4 | 4 | 5 | 3 | 4 |
| originality | 4 | 3 | 4 | 3 | 3 | 4 | 5 | 3 | 4 |
| clarity | 4 | 4 | 4 | 3 | 4 | 3 | 4 | 3 | 4 |
| specificity | 4 | 4 | 4 | 3 | 3 | 2 | 4 | 3 | 4 |
| 3.relevance | 5 | 4 | 4 | 3 | 4 | 4 | 5 | 3 | 3 |
| originality | 4 | 4 | 4 | 4 | 3 | 3 | 5 | 3 | 3 |
| clarity | 4 | 4 | 5 | 3 | 4 | 3 | 4 | 3 | 3 |
| specificity | 4 | 4 | 4 | 3 | 3 | 3 | 4 | 3 | 3 |
| 4.relevance | 5 | 5 | 4 | 3 | 4 | 4 | 5 | 1 | 4 |
| originality | 5 | 4 | 5 | 4 | 3 | 2 | 5 | 1 | 4 |
| clarity | 4 | 4 | 4 | 3 | 4 | 3 | 4 | 1 | 4 |
| specificity | 4 | 4 | 4 | 3 | 3 | 2 | 4 | 1 | 4 |
| 5.relevance | 5 | 5 | 4 | 3 | 3 | 4 | 5 | 2 | 3 |
| originality | 5 | 5 | 5 | 3 | 3 | 3 | 5 | 2 | 3 |
| clarity | 5 | 5 | 4 | 3 | 3 | 3 | 4 | 2 | 3 |
| specificity | 4 | 5 | 4 | 3 | 3 | 2 | 4 | 2 | 3 |
| Applications in other fields（其他领域的应用） | -2 | -2 | -2 | -2 | -2 | -2 | 1 | -2 | -2 |
| 1.relevance | 5 | 4 | 4 | 3 | 3 | 4 | 5 | 1 | 3 |
| originality | 5 | 5 | 4 | 3 | 3 | 4 | 5 | 1 | 3 |
| clarity | 5 | 4 | 4 | 3 | 3 | 3 | 4 | 1 | 3 |
| specificity | 4 | 4 | 4 | 3 | 3 | 4 | 4 | 1 | 3 |
| 2.relevance | 5 | 4 | 4 | 3 | 3 | 4 | 5 | 2 | 2 |
| originality | 5 | 5 | 5 | 4 | 3 | 3 | 5 | 2 | 2 |
| clarity | 5 | 5 | 4 | 3 | 3 | 3 | 5 | 2 | 2 |
| specificity | 5 | 5 | 5 | 3 | 3 | 3 | 4 | 2 | 2 |
| 3.relevance | 5 | 5 | 4 | 4 | 3 | 4 | 5 | 3 | 4 |
| originality | 4 | 5 | 5 | 3 | 3 | 4 | 5 | 3 | 4 |
| clarity | 4 | 4 | 4 | 3 | 3 | 3 | 5 | 3 | 4 |
| specificity | 5 | 4 | 5 | 3 | 3 | 3 | 4 | 3 | 4 |
| 4.relevance | 5 | 5 | 5 | 3 | 4 | 4 | 5 | 3 | 3 |
| originality | 4 | 5 | 4 | 3 | 4 | 2 | 5 | 3 | 3 |
| clarity | 4 | 5 | 4 | 3 | 4 | 3 | 5 | 3 | 3 |
| specificity | 5 | 5 | 4 | 3 | 3 | 3 | 4 | 3 | 3 |
| 5.relevance | 4 | 4 | 4 | 3 | 3 | 4 | 5 | 1 | 3 |
| originality | 5 | 5 | 4 | 3 | 3 | 2 | 5 | 1 | 3 |
| clarity | 5 | 3 | 4 | 3 | 2 | 3 | 5 | 1 | 3 |
| specificity | 5 | 3 | 4 | 3 | 2 | 3 | 4 | 1 | 3 |
| Total score | 434 | 430 | 437 | 350 | 316 | 317 | 449 | 220 | 316 |
