# Supplementary material for: Assessing the feasibility of large language models to identify top research priorities in enhanced external counterpulsation
Source: PLoS One. 2025 Apr 15;20(4):e0305442. doi: 10.1371/journal.pone.0305442 (PMC11999140; doi:10.1371/journal.pone.0305442)
Supplement: S1 File — (ZIP) [file pone.0305442.s001.zip › raw data and results --- ChatGPT 2.docx]

**应用大数据语言模型确定体外反搏研究重点”\n--- ChatGPT部分**

the principles of enhanced external counterpulsation（体外反搏的机制） [量表题]

**本题平均分：**1

| 选项 | 小计 | 比例 |
| --- | --- | --- |
| 1 | 1 | 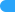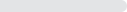11.11% |
| (空) | 8 | 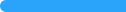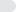88.89% |
| 本题有效填写人次 | 9 |  |

Integrated Insights into EECP Effects: Unveiling Hemodynamic, Neuroendocrine, and Inflammatory Responses and Their Implications for Cardiovascular and Systemic Health.综合洞察EECP效果：揭示血流动力学、神经内分泌和炎症反应及其对心血管和整体健康的影响 [矩阵量表题]

**该矩阵题平均分：3.81**

| 题目\选项 | 1 | 2 | 3 | 4 | 5 | 平均分 |
| --- | --- | --- | --- | --- | --- | --- |
| relevance | 0(0%) | 1(11.11%) | 1(11.11%) | 2(22.22%) | 5(55.56%) | 4.22 |
| originality | 0(0%) | 2(22.22%) | 1(11.11%) | 2(22.22%) | 4(44.44%) | 3.89 |
| clarity | 0(0%) | 1(11.11%) | 4(44.44%) | 2(22.22%) | 2(22.22%) | 3.56 |
| specificity | 0(0%) | 2(22.22%) | 2(22.22%) | 3(33.33%) | 2(22.22%) | 3.56 |
| 小计 | 0(0%) | 6(16.67%) | 8(22.22%) | 9(25%) | 13(36.11%) | 3.81 |

How does EECP compare to other treatment modalities such as pharmacotherapy, invasive procedures, or lifestyle interventions?EECP与其他治疗方式如药物治疗、侵入性手术或生活方式干预的比较 [矩阵量表题]

**该矩阵题平均分：3.47**

| 题目\选项 | 1 | 2 | 3 | 4 | 5 | 平均分 |
| --- | --- | --- | --- | --- | --- | --- |
| relevance | 0(0%) | 1(11.11%) | 2(22.22%) | 4(44.44%) | 2(22.22%) | 3.78 |
| originality | 0(0%) | 2(22.22%) | 2(22.22%) | 3(33.33%) | 2(22.22%) | 3.56 |
| clarity | 0(0%) | 1(11.11%) | 5(55.56%) | 2(22.22%) | 1(11.11%) | 3.33 |
| specificity | 0(0%) | 1(11.11%) | 6(66.67%) | 1(11.11%) | 1(11.11%) | 3.22 |
| 小计 | 0(0%) | 5(13.89%) | 15(41.67%) | 10(27.78%) | 6(16.67%) | 3.47 |

Optimizing EECP Treatment: Investigating Timing and Frequency for Varied Patient Populations and Clinical Conditions优化EECP治疗：针对不同患者群体和临床条件研究时机和频率 [矩阵量表题]

**该矩阵题平均分：3.64**

| 题目\选项 | 1 | 2 | 3 | 4 | 5 | 平均分 |
| --- | --- | --- | --- | --- | --- | --- |
| relevance | 0(0%) | 1(11.11%) | 1(11.11%) | 4(44.44%) | 3(33.33%) | 4 |
| originality | 0(0%) | 1(11.11%) | 2(22.22%) | 4(44.44%) | 2(22.22%) | 3.78 |
| clarity | 0(0%) | 1(11.11%) | 4(44.44%) | 3(33.33%) | 1(11.11%) | 3.44 |
| specificity | 0(0%) | 1(11.11%) | 5(55.56%) | 2(22.22%) | 1(11.11%) | 3.33 |
| 小计 | 0(0%) | 4(11.11%) | 12(33.33%) | 13(36.11%) | 7(19.44%) | 3.64 |

Refining Patient Selection for Enhanced External Counterpulsation (EECP): Optimizing Outcomes Based on Demographics, Comorbidities, and Treatment Responsiveness完善EECP治疗的患者选择：基于人口统计、合并症和治疗反应优化结果 [矩阵量表题]

**该矩阵题平均分：3.47**

| 题目\选项 | 1 | 2 | 3 | 4 | 5 | 平均分 |
| --- | --- | --- | --- | --- | --- | --- |
| relevance | 0(0%) | 1(11.11%) | 3(33.33%) | 1(11.11%) | 4(44.44%) | 3.89 |
| originality | 0(0%) | 1(11.11%) | 4(44.44%) | 2(22.22%) | 2(22.22%) | 3.56 |
| clarity | 0(0%) | 1(11.11%) | 5(55.56%) | 2(22.22%) | 1(11.11%) | 3.33 |
| specificity | 0(0%) | 2(22.22%) | 5(55.56%) | 1(11.11%) | 1(11.11%) | 3.11 |
| 小计 | 0(0%) | 5(13.89%) | 17(47.22%) | 6(16.67%) | 8(22.22%) | 3.47 |

Assessing the Long-Term Impact of EECP Therapy: Prospective Studies on Cardiovascular Outcomes, Quality of Life, and Healthcare Utilization评估EECP治疗的长期影响：前瞻性研究心血管结局、生活质量和医疗资源利用 [矩阵量表题]

**该矩阵题平均分：3.75**

| 题目\选项 | 1 | 2 | 3 | 4 | 5 | 平均分 |
| --- | --- | --- | --- | --- | --- | --- |
| relevance | 0(0%) | 0(0%) | 2(22.22%) | 3(33.33%) | 4(44.44%) | 4.22 |
| originality | 0(0%) | 0(0%) | 3(33.33%) | 5(55.56%) | 1(11.11%) | 3.78 |
| clarity | 0(0%) | 0(0%) | 5(55.56%) | 4(44.44%) | 0(0%) | 3.44 |
| specificity | 0(0%) | 0(0%) | 5(55.56%) | 3(33.33%) | 1(11.11%) | 3.56 |
| 小计 | 0(0%) | 0(0%) | 15(41.67%) | 15(41.67%) | 6(16.67%) | 3.75 |

Instrument improvements（结构改良） [量表题]

**本题平均分：**1

| 选项 | 小计 | 比例 |
| --- | --- | --- |
| 选项1 | 1 | 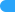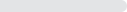11.11% |
| (空) | 8 | 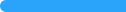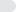88.89% |
| 本题有效填写人次 | 9 |  |

Revolutionizing EECP Treatment: Evaluating Compact and Portable Devices for Home-Based and Ambulatory Care, Integrating Technological Advancements for Enhanced EfficacyEECP治疗的革新：评估家用和移动护理的紧凑便携设备，整合技术提升治疗效果 [矩阵量表题]

**该矩阵题平均分：3.22**

| 题目\选项 | 1 | 2 | 3 | 4 | 5 | 平均分 |
| --- | --- | --- | --- | --- | --- | --- |
| relevance | 1(11.11%) | 1(11.11%) | 2(22.22%) | 3(33.33%) | 2(22.22%) | 3.44 |
| originality | 1(11.11%) | 1(11.11%) | 1(11.11%) | 4(44.44%) | 2(22.22%) | 3.56 |
| clarity | 1(11.11%) | 2(22.22%) | 2(22.22%) | 4(44.44%) | 0(0%) | 3 |
| specificity | 1(11.11%) | 3(33.33%) | 1(11.11%) | 4(44.44%) | 0(0%) | 2.89 |
| 小计 | 4(11.11%) | 7(19.44%) | 6(16.67%) | 15(41.67%) | 4(11.11%) | 3.22 |

Advancements in EECP Technology: Improving Treatment Efficacy and Patient Experience through Compact and Portable Devices, Automated Pressure Control, and Real-Time MonitoringEECP技术的进展：通过紧凑便携设备、自动压力控制和实时监测提升治疗效果与患者体验 [矩阵量表题]

**该矩阵题平均分：3.42**

| 题目\选项 | 1 | 2 | 3 | 4 | 5 | 平均分 |
| --- | --- | --- | --- | --- | --- | --- |
| relevance | 0(0%) | 2(22.22%) | 2(22.22%) | 2(22.22%) | 3(33.33%) | 3.67 |
| originality | 0(0%) | 2(22.22%) | 1(11.11%) | 4(44.44%) | 2(22.22%) | 3.67 |
| clarity | 0(0%) | 3(33.33%) | 3(33.33%) | 2(22.22%) | 1(11.11%) | 3.11 |
| specificity | 0(0%) | 3(33.33%) | 2(22.22%) | 3(33.33%) | 1(11.11%) | 3.22 |
| 小计 | 0(0%) | 10(27.78%) | 8(22.22%) | 11(30.56%) | 7(19.44%) | 3.42 |

Enhancing Patient Experience in EECP Treatment: Investigating Ergonomic Enhancements for Adjustable Seating, Cushioning, and Noise ReductionEECP治疗中患者体验的提升：探索可调座位、缓冲材料和降噪功能的人体工程学改进 [矩阵量表题]

**该矩阵题平均分：3.92**

| 题目\选项 | 1 | 2 | 3 | 4 | 5 | 平均分 |
| --- | --- | --- | --- | --- | --- | --- |
| relevance | 0(0%) | 0(0%) | 1(11.11%) | 6(66.67%) | 2(22.22%) | 4.11 |
| originality | 0(0%) | 0(0%) | 2(22.22%) | 6(66.67%) | 1(11.11%) | 3.89 |
| clarity | 0(0%) | 0(0%) | 2(22.22%) | 6(66.67%) | 1(11.11%) | 3.89 |
| specificity | 0(0%) | 0(0%) | 3(33.33%) | 5(55.56%) | 1(11.11%) | 3.78 |
| 小计 | 0(0%) | 0(0%) | 8(22.22%) | 23(63.89%) | 5(13.89%) | 3.92 |

Integrating EECP Devices with Telemedicine and Mobile Health: Enhancing Remote Monitoring and Patient Care将EECP设备与远程医疗和移动健康平台整合：增强远程监测和患者护理 [矩阵量表题]

**该矩阵题平均分：3.61**

| 题目\选项 | 1 | 2 | 3 | 4 | 5 | 平均分 |
| --- | --- | --- | --- | --- | --- | --- |
| relevance | 0(0%) | 0(0%) | 4(44.44%) | 2(22.22%) | 3(33.33%) | 3.89 |
| originality | 0(0%) | 0(0%) | 4(44.44%) | 4(44.44%) | 1(11.11%) | 3.67 |
| clarity | 0(0%) | 0(0%) | 5(55.56%) | 3(33.33%) | 1(11.11%) | 3.56 |
| specificity | 0(0%) | 1(11.11%) | 5(55.56%) | 2(22.22%) | 1(11.11%) | 3.33 |
| 小计 | 0(0%) | 1(2.78%) | 18(50%) | 11(30.56%) | 6(16.67%) | 3.61 |

Optimizing EECP Treatment: Investigating Safety Mechanisms for Enhanced Patient Security优化EECP治疗：探索安全机制以提升患者安全性 [矩阵量表题]

**该矩阵题平均分：3.44**

| 题目\选项 | 1 | 2 | 3 | 4 | 5 | 平均分 |
| --- | --- | --- | --- | --- | --- | --- |
| relevance | 1(11.11%) | 0(0%) | 2(22.22%) | 3(33.33%) | 3(33.33%) | 3.78 |
| originality | 1(11.11%) | 1(11.11%) | 2(22.22%) | 2(22.22%) | 3(33.33%) | 3.56 |
| clarity | 1(11.11%) | 0(0%) | 4(44.44%) | 3(33.33%) | 1(11.11%) | 3.33 |
| specificity | 1(11.11%) | 1(11.11%) | 3(33.33%) | 4(44.44%) | 0(0%) | 3.11 |
| 小计 | 4(11.11%) | 2(5.56%) | 11(30.56%) | 12(33.33%) | 7(19.44%) | 3.44 |

in the field of heart disease（在心血管领域的应用） [量表题]

**本题平均分：**1

| 选项 | 小计 | 比例 |
| --- | --- | --- |
| 选项1 | 1 | 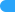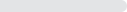11.11% |
| (空) | 8 | 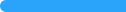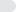88.89% |
| 本题有效填写人次 | 9 |  |

Unlocking Vascular Relaxation: Investigating Signaling Pathways in EECP Treatment through eNOS, cGMP, and Vasodilatory Factors解锁血管松弛：通过eNOS、cGMP和扩血管因子探讨EECP治疗中的信号通路 [矩阵量表题]

**该矩阵题平均分：3.72**

| 题目\选项 | 1 | 2 | 3 | 4 | 5 | 平均分 |
| --- | --- | --- | --- | --- | --- | --- |
| relevance | 0(0%) | 0(0%) | 3(33.33%) | 4(44.44%) | 2(22.22%) | 3.89 |
| originality | 0(0%) | 1(11.11%) | 4(44.44%) | 1(11.11%) | 3(33.33%) | 3.67 |
| clarity | 0(0%) | 0(0%) | 4(44.44%) | 2(22.22%) | 3(33.33%) | 3.89 |
| specificity | 0(0%) | 1(11.11%) | 4(44.44%) | 3(33.33%) | 1(11.11%) | 3.44 |
| 小计 | 0(0%) | 2(5.56%) | 15(41.67%) | 10(27.78%) | 9(25%) | 3.72 |

Unveiling the Future of Cardiac Health: Investigating EECP Treatment Integration with Telemedicine and Mobile Health for Remote Monitoring揭示心脏健康的未来：通过远程监测探讨EECP治疗与远程医疗和移动健康的整合 [矩阵量表题]

**该矩阵题平均分：3.61**

| 题目\选项 | 1 | 2 | 3 | 4 | 5 | 平均分 |
| --- | --- | --- | --- | --- | --- | --- |
| relevance | 0(0%) | 0(0%) | 4(44.44%) | 3(33.33%) | 2(22.22%) | 3.78 |
| originality | 0(0%) | 0(0%) | 4(44.44%) | 3(33.33%) | 2(22.22%) | 3.78 |
| clarity | 0(0%) | 0(0%) | 5(55.56%) | 3(33.33%) | 1(11.11%) | 3.56 |
| specificity | 0(0%) | 1(11.11%) | 4(44.44%) | 4(44.44%) | 0(0%) | 3.33 |
| 小计 | 0(0%) | 1(2.78%) | 17(47.22%) | 13(36.11%) | 5(13.89%) | 3.61 |

Longitudinal Assessment of EECP Impact on Endothelial Function, Progenitor Cell Activity, and Vascular Inflammation: Implications for Cardiovascular Disease Prevention and Management纵向评估EECP对内皮功能、内皮祖细胞活性和血管炎症标志物的影响：对心血管疾病预防和管理的启示 [矩阵量表题]

**该矩阵题平均分：3.78**

| 题目\选项 | 1 | 2 | 3 | 4 | 5 | 平均分 |
| --- | --- | --- | --- | --- | --- | --- |
| relevance | 0(0%) | 1(11.11%) | 0(0%) | 6(66.67%) | 2(22.22%) | 4 |
| originality | 0(0%) | 1(11.11%) | 2(22.22%) | 4(44.44%) | 2(22.22%) | 3.78 |
| clarity | 0(0%) | 1(11.11%) | 2(22.22%) | 5(55.56%) | 1(11.11%) | 3.67 |
| specificity | 0(0%) | 1(11.11%) | 2(22.22%) | 5(55.56%) | 1(11.11%) | 3.67 |
| 小计 | 0(0%) | 4(11.11%) | 6(16.67%) | 20(55.56%) | 6(16.67%) | 3.78 |

Unraveling Dynamic Hemodynamic Responses: Investigating Cardiac Output, Systemic Vascular Resistance Changes, and Implications for Non-Pharmacological Heart Failure Management in EECP Treatment揭示动态血流动力学响应：探究EECP治疗中心输出、系统血管阻力变化及其对非药物性心力衰竭管理的意义 [矩阵量表题]

**该矩阵题平均分：3.94**

| 题目\选项 | 1 | 2 | 3 | 4 | 5 | 平均分 |
| --- | --- | --- | --- | --- | --- | --- |
| relevance | 0(0%) | 0(0%) | 2(22.22%) | 4(44.44%) | 3(33.33%) | 4.11 |
| originality | 0(0%) | 1(11.11%) | 2(22.22%) | 2(22.22%) | 4(44.44%) | 4 |
| clarity | 0(0%) | 0(0%) | 4(44.44%) | 2(22.22%) | 3(33.33%) | 3.89 |
| specificity | 0(0%) | 1(11.11%) | 3(33.33%) | 2(22.22%) | 3(33.33%) | 3.78 |
| 小计 | 0(0%) | 2(5.56%) | 11(30.56%) | 10(27.78%) | 13(36.11%) | 3.94 |

Examining Autonomic Regulation: HRV Parameters, Sympathetic-Parasympathetic Balance, and Baroreflex Sensitivity Dynamics Following EECP Treatment评估自主调节：EECP治疗后心率变异性参数、交感-副交感平衡和压力反射敏感性动态 [矩阵量表题]

**该矩阵题平均分：3.42**

| 题目\选项 | 1 | 2 | 3 | 4 | 5 | 平均分 |
| --- | --- | --- | --- | --- | --- | --- |
| relevance | 1(11.11%) | 0(0%) | 2(22.22%) | 3(33.33%) | 3(33.33%) | 3.78 |
| originality | 1(11.11%) | 2(22.22%) | 1(11.11%) | 3(33.33%) | 2(22.22%) | 3.33 |
| clarity | 1(11.11%) | 2(22.22%) | 1(11.11%) | 3(33.33%) | 2(22.22%) | 3.33 |
| specificity | 1(11.11%) | 1(11.11%) | 2(22.22%) | 5(55.56%) | 0(0%) | 3.22 |
| 小计 | 4(11.11%) | 5(13.89%) | 6(16.67%) | 14(38.89%) | 7(19.44%) | 3.42 |

in the field of neurology（在神经内科领域的应用） [量表题]

**本题平均分：**1

| 选项 | 小计 | 比例 |
| --- | --- | --- |
| 选项1 | 1 | 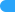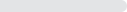11.11% |
| (空) | 8 | 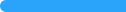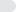88.89% |
| 本题有效填写人次 | 9 |  |

Neuroimaging Insights into Structural and Functional Changes Induced by EECP: Implications for Neural Repair and Recovery Processes.利用先进的神经影像技术评估EECP诱导的结构变化（例如，灰质体积、白质完整性）和功能连接的改变，探究其对神经修复和恢复过程的意义 [矩阵量表题]

**该矩阵题平均分：3.83**

| 题目\选项 | 1 | 2 | 3 | 4 | 5 | 平均分 |
| --- | --- | --- | --- | --- | --- | --- |
| relevance | 0(0%) | 0(0%) | 2(22.22%) | 4(44.44%) | 3(33.33%) | 4.11 |
| originality | 0(0%) | 1(11.11%) | 3(33.33%) | 2(22.22%) | 3(33.33%) | 3.78 |
| clarity | 0(0%) | 0(0%) | 3(33.33%) | 4(44.44%) | 2(22.22%) | 3.89 |
| specificity | 0(0%) | 1(11.11%) | 3(33.33%) | 4(44.44%) | 1(11.11%) | 3.56 |
| 小计 | 0(0%) | 2(5.56%) | 11(30.56%) | 14(38.89%) | 9(25%) | 3.83 |

Synergistic Approaches to Neurological Rehabilitation: Investigating the Combined Effects of EECP with Physical, Occupational, and Cognitive Interventions on Motor and Cognitive Recovery Post-Stroke神经康复的协同方法：研究EECP与物理治疗、职业治疗和认知康复干预的综合效应，促进中风后的运动和认知恢复 [矩阵量表题]

**该矩阵题平均分：3.69**

| 题目\选项 | 1 | 2 | 3 | 4 | 5 | 平均分 |
| --- | --- | --- | --- | --- | --- | --- |
| relevance | 0(0%) | 0(0%) | 2(22.22%) | 5(55.56%) | 2(22.22%) | 4 |
| originality | 0(0%) | 0(0%) | 4(44.44%) | 4(44.44%) | 1(11.11%) | 3.67 |
| clarity | 0(0%) | 0(0%) | 3(33.33%) | 6(66.67%) | 0(0%) | 3.67 |
| specificity | 0(0%) | 1(11.11%) | 3(33.33%) | 5(55.56%) | 0(0%) | 3.44 |
| 小计 | 0(0%) | 1(2.78%) | 12(33.33%) | 20(55.56%) | 3(8.33%) | 3.69 |

Enhancing Neurological Recovery: Assessing the Impact of EECP on Cognitive Function, Neuroplasticity, and Rehabilitation Potential Following Traumatic Brain Injury增强神经恢复：评估EECP对创伤性脑损伤后认知功能、神经可塑性和康复潜力的影响 [矩阵量表题]

**该矩阵题平均分：3.67**

| 题目\选项 | 1 | 2 | 3 | 4 | 5 | 平均分 |
| --- | --- | --- | --- | --- | --- | --- |
| relevance | 0(0%) | 0(0%) | 3(33.33%) | 4(44.44%) | 2(22.22%) | 3.89 |
| originality | 0(0%) | 0(0%) | 4(44.44%) | 4(44.44%) | 1(11.11%) | 3.67 |
| clarity | 0(0%) | 0(0%) | 4(44.44%) | 4(44.44%) | 1(11.11%) | 3.67 |
| specificity | 0(0%) | 0(0%) | 5(55.56%) | 4(44.44%) | 0(0%) | 3.44 |
| 小计 | 0(0%) | 0(0%) | 16(44.44%) | 16(44.44%) | 4(11.11%) | 3.67 |

The Impact of Enhanced External Counterpulsation on Neurovascular Coupling and Cerebral Perfusion: Investigating Mechanisms and Potential Therapeutic Applications in Neurovascular Disorders增强外部反搏对神经血管耦合和脑灌注的影响：探讨机制及在神经血管疾病中的潜在治疗应用 [矩阵量表题]

**该矩阵题平均分：3.56**

| 题目\选项 | 1 | 2 | 3 | 4 | 5 | 平均分 |
| --- | --- | --- | --- | --- | --- | --- |
| relevance | 1(11.11%) | 0(0%) | 1(11.11%) | 4(44.44%) | 3(33.33%) | 3.89 |
| originality | 1(11.11%) | 1(11.11%) | 1(11.11%) | 3(33.33%) | 3(33.33%) | 3.67 |
| clarity | 1(11.11%) | 0(0%) | 2(22.22%) | 6(66.67%) | 0(0%) | 3.44 |
| specificity | 1(11.11%) | 1(11.11%) | 2(22.22%) | 5(55.56%) | 0(0%) | 3.22 |
| 小计 | 4(11.11%) | 2(5.56%) | 6(16.67%) | 18(50%) | 6(16.67%) | 3.56 |

Exploring the Neurotrophic and Neuroregenerative Potential of EECP: Implications for Disease Pathology, Cognitive Decline, and Motor Symptoms探究EECP的神经营养和神经再生潜力：对疾病病理、认知衰退和运动症状的影响的意义 [矩阵量表题]

**该矩阵题平均分：3.61**

| 题目\选项 | 1 | 2 | 3 | 4 | 5 | 平均分 |
| --- | --- | --- | --- | --- | --- | --- |
| relevance | 0(0%) | 1(11.11%) | 3(33.33%) | 2(22.22%) | 3(33.33%) | 3.78 |
| originality | 0(0%) | 1(11.11%) | 4(44.44%) | 0(0%) | 4(44.44%) | 3.78 |
| clarity | 0(0%) | 1(11.11%) | 4(44.44%) | 2(22.22%) | 2(22.22%) | 3.56 |
| specificity | 0(0%) | 2(22.22%) | 3(33.33%) | 3(33.33%) | 1(11.11%) | 3.33 |
| 小计 | 0(0%) | 5(13.89%) | 14(38.89%) | 7(19.44%) | 10(27.78%) | 3.61 |

Applications in other fields（其他领域的应用） [量表题]

**本题平均分：**1

| 选项 | 小计 | 比例 |
| --- | --- | --- |
| 选项1 | 1 | 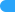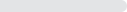11.11% |
| (空) | 8 | 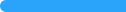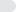88.89% |
| 本题有效填写人次 | 9 |  |

Optimizing Orthopedic Wellness: Evaluating the Impact of EECP on Joint Function, Muscle Strength, and Pain Management in Osteoarthritis, Rheumatoid Arthritis, and Musculoskeletal Injuries优化骨科健康：评估EECP对骨关节炎、类风湿性关节炎和肌肉骨骼损伤患者关节功能、肌肉力量和疼痛管理的影响 [矩阵量表题]

**该矩阵题平均分：3.47**

| 题目\选项 | 1 | 2 | 3 | 4 | 5 | 平均分 |
| --- | --- | --- | --- | --- | --- | --- |
| relevance | 1(11.11%) | 0(0%) | 3(33.33%) | 3(33.33%) | 2(22.22%) | 3.56 |
| originality | 1(11.11%) | 0(0%) | 3(33.33%) | 2(22.22%) | 3(33.33%) | 3.67 |
| clarity | 1(11.11%) | 0(0%) | 4(44.44%) | 3(33.33%) | 1(11.11%) | 3.33 |
| specificity | 1(11.11%) | 0(0%) | 3(33.33%) | 5(55.56%) | 0(0%) | 3.33 |
| 小计 | 4(11.11%) | 0(0%) | 13(36.11%) | 13(36.11%) | 6(16.67%) | 3.47 |

Unveiling the Metabolic Impact of EECP: Exploring Effects on Insulin Sensitivity, Lipid Profiles, and Inflammatory Markers for Managing Metabolic Syndrome and Cardiovascular Risk Factors揭示EECP的代谢影响：探究其对胰岛素敏感性、血脂和炎症标志物的影响，以管理代谢综合征和降低心血管危险因素 [矩阵量表题]

**该矩阵题平均分：3.61**

| 题目\选项 | 1 | 2 | 3 | 4 | 5 | 平均分 |
| --- | --- | --- | --- | --- | --- | --- |
| relevance | 0(0%) | 2(22.22%) | 2(22.22%) | 3(33.33%) | 2(22.22%) | 3.56 |
| originality | 0(0%) | 2(22.22%) | 2(22.22%) | 1(11.11%) | 4(44.44%) | 3.78 |
| clarity | 0(0%) | 2(22.22%) | 3(33.33%) | 1(11.11%) | 3(33.33%) | 3.56 |
| specificity | 0(0%) | 2(22.22%) | 3(33.33%) | 1(11.11%) | 3(33.33%) | 3.56 |
| 小计 | 0(0%) | 8(22.22%) | 10(27.78%) | 6(16.67%) | 12(33.33%) | 3.61 |

Enhancing Sleep Quality with EECP: Exploring Effects on Sleep Architecture, Latency, and Efficiency as a Non-Pharmacological Intervention for Sleep Disorders利用EECP提高睡眠质量：探究其对睡眠结构、潜伏期和效率的影响，作为睡眠障碍的非药物干预措施 [矩阵量表题]

**该矩阵题平均分：3.89**

| 题目\选项 | 1 | 2 | 3 | 4 | 5 | 平均分 |
| --- | --- | --- | --- | --- | --- | --- |
| relevance | 0(0%) | 0(0%) | 2(22.22%) | 4(44.44%) | 3(33.33%) | 4.11 |
| originality | 0(0%) | 0(0%) | 3(33.33%) | 3(33.33%) | 3(33.33%) | 4 |
| clarity | 0(0%) | 0(0%) | 4(44.44%) | 4(44.44%) | 1(11.11%) | 3.67 |
| specificity | 0(0%) | 0(0%) | 4(44.44%) | 3(33.33%) | 2(22.22%) | 3.78 |
| 小计 | 0(0%) | 0(0%) | 13(36.11%) | 14(38.89%) | 9(25%) | 3.89 |

Renal Rejuvenation: Investigating the Effects of EECP on Renal Blood Flow, Glomerular Filtration Rate, and Kidney Injury Markers for Managing Renal Artery Stenosis and Acute Kidney Injury肾脏复苏：研究EECP对肾血流、肾小球滤过率和肾损伤标志物的影响，以管理肾动脉狭窄和急性肾损伤 [矩阵量表题]

**该矩阵题平均分：3.81**

| 题目\选项 | 1 | 2 | 3 | 4 | 5 | 平均分 |
| --- | --- | --- | --- | --- | --- | --- |
| relevance | 0(0%) | 0(0%) | 3(33.33%) | 2(22.22%) | 4(44.44%) | 4.11 |
| originality | 0(0%) | 1(11.11%) | 3(33.33%) | 3(33.33%) | 2(22.22%) | 3.67 |
| clarity | 0(0%) | 0(0%) | 4(44.44%) | 3(33.33%) | 2(22.22%) | 3.78 |
| specificity | 0(0%) | 0(0%) | 5(55.56%) | 2(22.22%) | 2(22.22%) | 3.67 |
| 小计 | 0(0%) | 1(2.78%) | 15(41.67%) | 10(27.78%) | 10(27.78%) | 3.81 |

Utilizing Enhanced External Counterpulsation to Enhance Cancer Treatment Outcomes: Investigating Chemotherapy Delivery, Cardiotoxicity Reduction, and Treatment Efficacy Enhancement利用增强外部反搏改善癌症治疗效果：探讨化疗药物输送、减少治疗相关心毒性和提高整体治疗效果的潜力 [矩阵量表题]

**该矩阵题平均分：3.31**

| 题目\选项 | 1 | 2 | 3 | 4 | 5 | 平均分 |
| --- | --- | --- | --- | --- | --- | --- |
| relevance | 1(11.11%) | 0(0%) | 3(33.33%) | 4(44.44%) | 1(11.11%) | 3.44 |
| originality | 1(11.11%) | 1(11.11%) | 3(33.33%) | 1(11.11%) | 3(33.33%) | 3.44 |
| clarity | 1(11.11%) | 1(11.11%) | 4(44.44%) | 1(11.11%) | 2(22.22%) | 3.22 |
| specificity | 1(11.11%) | 1(11.11%) | 4(44.44%) | 2(22.22%) | 1(11.11%) | 3.11 |
| 小计 | 4(11.11%) | 3(8.33%) | 14(38.89%) | 8(22.22%) | 7(19.44%) | 3.31 |

**题目平均分之和：367.67**
