# Supplementary material for: Assessing the feasibility of large language models to identify top research priorities in enhanced external counterpulsation
Source: PLoS One. 2025 Apr 15;20(4):e0305442. doi: 10.1371/journal.pone.0305442 (PMC11999140; doi:10.1371/journal.pone.0305442)
Supplement: Attachment — Submitted filename: Response to Reviewers.docx [file pone.0305442.s003.docx]

**Responses to Reviewer Comments**

The authors would like to express our gratitude to the editor for the opportunity to revise our paper. We also extend our thanks to the reviewers for their comments, which have significantly improved the manuscript. For details, please refer to the following responses (reviewer comments are in italics, while our responses are in regular text).

**Reviewer #1:**

1、*In the paper, some figures in the manuscript are a little blurry, please improve the clarity.*

**Authors' Response:**

Reviewer 1 suggested enhancing the clarity of the figures. We have improved the clarity of the figures in the revised manuscript.

2、*Since there are some papers in this topic, the contributions of the manuscript should be better summarized and listed.*

**Authors' Response:**

We fully agree with the reviewers' suggestions and have implemented the recommended changes in the manuscript. We have summarized the contributions of our paper, specifically highlighted in the discussion section, where the relevant text is marked in red. (Please refer to the text highlighted in red on page 18, lines 291 to 300)

*3、While the introduction sets the context, a more explicit literature review section could better situate the study within the broader research landscape. I also recommend considering these paper, such as Evaluating the Use of Large Language Model, LESSON Multi-Label Adversarial False Data Injection Attack for Deep Learning Locational Detection, Application Research of Large Language Models in Medicine: Status, Problems, and Future and so on. These references could provide valuable insights into your research.*

**Authors' Response:**

We sincerely appreciate your valuable feedback. After re-examining the relevant literature, we have revised the section in line with your suggestions. Additionally, we have incorporated references to "Application Research of Large Language Models in Medicine: Status, Problems, and Future. IEEE" and "Evaluating the Use of Large Language Models in Identifying Top Research Questions in Gastroenterology. Scientific Reports." into the Introduction section of the revised manuscript (Please refer to the text highlighted in red on page 2, lines 302 to 313)

8.Ao G, Wang G, Chen Y, Xia J, Su S. Application research of large language models in medicine: status, problems, and future. In: 2023 13th International Conference on Information Technology in Medicine and Education. IEEE; 2023. p. 734–739. Available from: https://doi.org/10.1109/ITME60234.2023.00151.

9.Lahat A, Shachar E, Avidan B, Shatz Z, Glicksberg BS, Klang E. Evaluating the use of large language model in identifying top research questions in gastroenterology. Sci Rep. 2023; 13(1): 4164. https://doi.org/10.1038/s41598-023-31412-2

*4、Add a section on the limitations of the work and future work in this paper.*

**Authors' Response:**

Thank you for your valuable and inspiring suggestions. Based on your feedback, we have added a paragraph detailing the limitations. In this paragraph, we systematically outline the constraints of our work, including the limitations of the expert panels, potential subjective rating bias, and models may lack access to the last-but-one paragraph literature.（Please refer to the text highlighted in red on page 18, lines 52 to 60 ）

*5、The manuscript contains a number of linguistic errors that hinder comprehension. The authors are advised to make careful revisions and improvements.*

**Authors' Response:**

We sincerely thank the reviewer for their careful reading. We apologize for the careless mistakes and have made every effort to improve the manuscript. In the resubmitted manuscript, we have meticulously corrected all identified typos. This includes a thorough review of the entire document to ensure no errors were overlooked. Thank you for your detailed attention to this issue, which has helped us improve the quality of our work.

**Reviewer #2:** *This study tackles an interesting and emerging topic that use powerful LLMs to identify top research priorities in EECP. It is well known that ChatGPT was trained on general text and it is not meant to solve complex deep domain-specific problems. The main conclusion derived in this study is expected. The reviewer is not capable of judging the soundness of the generated research priorities. The ranking from each selected expert is meaningful. However, their aggregation may not as the rankings of different experts may not comparable. Hence, the mean/std in Table 5 may not be very meaningful*.

**Authors' Response:**

We sincerely appreciate your professional review of our article. We would like to emphasize that our expert team consists of Associate Directors and/or Chief Physicians, each with over 20 years of extensive experience in EECP. They have gained their expertise through clinical practice and have made significant contributions to academic research, with each expert having published at least five scholarly articles related to EECP. Furthermore, they have actively contributed to the development of guidelines in the EECP field, playing a crucial role in its standardized advancement. Given their extensive knowledge, our expert team is well-equipped to assess our research priorities across four parameters—relevance, originality, clarity, and specificity—using a scoring scale from 1 to 5.

To compile assessment results from all experts and derive meaningful insights, we created three scoring tables for each large language model, with evaluation indicators, evaluators, and topics serving as column attributes. The results presented in Tables 3 to 5 are derived from descriptive statistical methods applied to these tables. Additionally, to accurately represent potential non-normal distributions, we included quartiles alongside mean values and standard deviations in Tables 3 to 5. This approach has effectively minimized the influence of extreme values that may not accurately reflect reality, making the rankings from different experts much more comparable.
