# Supplementary material for: Assessing the feasibility of large language models to identify top research priorities in enhanced external counterpulsation
Source: PLoS One. 2025 Apr 15;20(4):e0305442. doi: 10.1371/journal.pone.0305442 (PMC11999140; doi:10.1371/journal.pone.0305442)
Supplement: Attachment — Submitted filename: Responses to Reviewer Comments.docx [file pone.0305442.s004.docx]

**Responses to Reviewer Comments**

The authors would like to express our gratitude to the editor for the opportunity to revise our paper. We also extend our thanks to the reviewers for their comments, which have significantly improved the manuscript. For details, please refer to the following responses (reviewer comments are in italics, while our responses are in regular text).

**Reviewer**

*It is very important that the authors provide evidence to support their "Expert Evaluation". Please provide the detail as annex to proceed further.*

**Authors' Response:**

We sincerely appreciate your professional review of our article. Based on your feedback,we have supplemented the expert profiles, which are detailed in the attachment entitled "Curriculum Vitae of Review Experts".Additionally, the raw data and results of the expert evaluation are provided in Appendix "raw data and results "for further reference.
